# Trends and patterns of North Korea’s disease burden from 1990 to 2019: Results from Global Burden of Disease study 2019

**DOI:** 10.1371/journal.pone.0277335

**Published:** 2022-11-14

**Authors:** Eun Hae Lee, Minjae Choi, Joshua Kirabo Sempungu, Yo Han Lee

**Affiliations:** 1 Department of Preventive Medicine, Korea University College of Medicine, Seoul, South Korea; 2 Institute for Future Public Health, Graduate School of Public Health, Korea University, Seoul, South Korea; 3 Graduate School of Medicine, Ajou University, Suwon, Gyeonggi-do, South Korea; Non-Communicable Diseases Research Center, Endocrinology and Metabolism Population Sciences Institute, Tehran University of Medical Sciences, ISLAMIC REPUBLIC OF IRAN

## Abstract

**Background:**

Evidence for the trends and patterns of disease burden in North Korea is limited, and in-depth analysis based on several health outcomes for a better understanding remains challenging. Therefore, we aimed to investigate the trends and patterns of disease burden in North Korea between 1990 and 2019.

**Methods:**

We used data from the Global Burden of Diseases (GBD) 2019 study to analyze the North Korean disease burden in comparison with four groups: global, South Korea, low-sociodemographic index (SDI) countries, Central and Eastern Europe, and Central Asia (former socialist countries). We also examined changes in the disease burden between 1990 and 2019 by disease category and age group.

**Findings:**

In 2019, in North Korea, death rates and disability-adjusted life years (DALYs) rates were reduced by 22.2% and 30.7%, respectively, compared to 1990. The rates showed similar trends and patterns to that in former socialist countries. However, these reductions were lower than those of the global rates and rates in low-SDI countries and South Korea. Death rates and DALY rates for under five years dramatically decreased by more than 78%, similar to the trend in South Korea. In contrast, the decline in the death rates and DALY rates of adults was less than those worldwide and in low-SDI countries and South Korea. The burden of diseases among those aged ≥30 years increased largely due to the persistently high burden of non-communicable diseases (NCDs). Cardiovascular diseases, neoplasms, and chronic respiratory diseases were the leading causes of the disease burden in both 1990 and 2019.

**Interpretation:**

North Korea’s disease burden patterns and trends show clear improvements over the past 30 years but suggest that the current challenges of NCDs in the country are very serious. NCDs should be no longer neglected and should be prioritized in public health agendas in North Korea.

## Introduction

The Democratic People’s Republic of Korea (hereafter referred to as North Korea) has experienced a series of geopolitical transitions, and economic difficulties since the 1990s, including natural disasters and the collapse of the Soviet Union, which contributed to a great famine called the “March of Hardship” in the mid-1990s [1]. Moreover, international communities such as the United Nations (UN) and the European Union (EU) have imposed economic sanctions against North Korea due to nuclear weapon tests over the past decades [2]. North Korea has also shown a demographic transition toward an aging population. The fertility rate in North Korea showed a decreasing trend from 2.3 in 1990 to 1.9 in 2019, and the proportion of the elderly population has been gradually increasing since 1990 [3]. It shows a similar tendency to South Korea where fertility rate is decreasing from 1.6 in 1990 to 0.9 in 2019, and the elderly population continues to increase to 14.9% of the total population aged 65 or older in 2019 [4]. These transitions have had a considerable impact on the economy and public health [5]. Inadequate healthcare and welfare services for individuals to prevent diseases and malnutrition due to economic hardships contribute to a high disease burden [6]. Moreover, the closed social structure of North Korea makes it difficult to improve the population’s health.

According to the Global Burden of Diseases (GBD) 2017 study, conducted by the Institute for Health Metrics and Evaluation (IHME), life expectancy in low-sociodemographic index (SDI) counties increased from 1990 (53.8 years) to 2017 (65.8 years), and life expectancy in Central and Eastern Europe, and Central Asian counties increased from 1990 (69.4 years) to 2017 (73.0 years), while life expectancy in North Korea remained almost unchanged from 1990 (71.8 years) to 2017 (72.0 years) [7]. The high death rates in North Korea in the past decades is largely due to communicable, maternal, neonatal, and nutritional diseases (CMNNDs), similar to the disease structure of low-income countries [8]. However, the current public health problems range from CMNNDs to non-communicable diseases (NCDs); therefore, the disease structure in North Korea should be considered not as a communicable disease structure [2, 9] but as a double burden of non-communicable and communicable diseases [8]. Thus, investigating disease trends and determining the priority of the various non-communicable and communicable diseases is necessary to develop national public health policies in North Korea.

However, evidence for the patterns and trends of the disease burden in North Korea is still unclear, and an in-depth analysis including a range of specific diseases using various health outcomes would provide a better understanding of the disease burden in North Korea. Furthermore, an accurate understanding of individual health status remains difficult due to the inadequacy and low quality of the epidemiological data [8]. Only a few studies have investigated the health status of North Koreans using data from international organizations, including the World Health Organization (WHO) [8, 10], but these studies are limited in scope and time frame and do not report a comprehensive comparison with other countries. Lee et al. [8] reported the overall disease burden of North Koreans using data from the WHO and suggested the need to handle both communicable and non-communicable diseases; however, the study was limited to a 1-year time frame with broad disease categories. Although Ha and Lee [10] made progress and highlighted the importance of the burden of NCDs, reporting trends of death rates in North Korea from 1960 to 2017, the study was also limited to broad disease categories (e.g., communicable diseases, cancer, and cardiovascular disease), with no comparison groups, and used the number of deaths as the only health outcome.

The GBD study provides reliable estimates for population health outcomes, such as incidence, prevalence, death rates, and disability-adjusted life years (DALYs) of 369 diseases across 204 countries, including North Korea [11], and is a key source of data to investigate adverse or progressive disease patterns in North Korea. In the current study, we aimed to investigate the trends and patterns of the disease burden in North Korea between 1990–2019 with data from the GBD 2019 study.

## Methods

### Overview of the GBD 2019 study

The GBD 2019 study uses comprehensive data and methods to compare the size of disease, injuries, and risk factors by age group, sex, country, region, and time. Global, regional, and national estimates for death rates, incidence, prevalence, years of life lost (YLLs), years of healthy life lost due to disability (YLDs), and DALY rates were published in 2019. In GBD 2019 study, the Cause of Death Ensemble model (CODEm) was used to estimate the age and cause-specific death rates. CODEm computes death rates estimates for each sex, age, and year through a standardized modeling process using out-of-sample efficacy analysis and covariant selection [11]. DisMod-MR is a Bayesian meta-regression tool that evaluates all available data for incidence, prevalence, and death rates, with enhanced consistency. DALY rates for premature death and disability are useful for quantifying and ranking health losses due to specific diseases and injuries. DALY rates help in understanding the main causes of health burdens and in identifying the existence of gaps over time [12]. The GBD study classified the causes of death into four- Level 1 causes, representing three main categories: CMNNDs, NCDs, and Injuries. Level 2 consists of 22 causes such as respiratory infections and tuberculosis, cardiovascular disease, and traffic injuries. Level 3 has 174 causes of disease, including specific causes such as tuberculosis, stroke, and road injuries, while Level 4 has 301 detailed categories. More information is available elsewhere [13].

### Data source

This study used estimates from the GBD 2019 to analyze the trends and patterns of the disease burden in North Korea. The IHME annually reports estimates for the disease burden and provides GBD data online. It can also be downloaded from Global Health Data Exchange [13]. Detailed descriptions of the metrics and analyses used in the GBD 2019 are reported elsewhere [11]. To estimate the disease burden in North Korea, GBD 2019 used 77 different data sources, including WHO STEPS survey data, United Nations Children’s Fund (UNICEF), World Food Program (WFP) nutrition assessment, Multi-indicator Cluster Survey (MICS), World Medicines Report, World Malaria Report, and undisclosed national documents of the North Korean government.

### Data presentation

All death rates and DALY rates values presented in this study were age-standardized rates per 100,000 people. The age standardization rate is the weighted average of the age-specific rate, where the weight is the rate of the standard population in the corresponding age group. The potential confounding effect of age is eliminated when comparing the calculated age-standardized rates using the same standard population. We present the trends in death rates and DALY rates between 1990 and 2019 for North Korea and four comparison groups by age group and level 1 causes with a 95% UI for each point estimate. To explain how death rates and DALY rates changed from 1990 to 2019, we presented the percentage changes. The four comparison groups are global, the Republic of Korea (hereafter referred to as South Korea), Central and Eastern Europe, and Central Asia (one of the seven super-regions of the GBD study, hereafter referred to as former socialist countries). Socialist countries refer to post-socialist countries of Eastern Europe and the former Soviet Union, including North Korea. These countries (including North Korea) have a low-sociodemographic index (SDI), a composite measure of sociodemographic development status. The SDI was calculated based on per capita income, average education level (population aged ≥15 years), and the total fertility rate of women under 25 years. The higher the value, the higher the level of development, and it is divided into five groups. We compared death rates and DALY rates for different age groups (<1 year, under 5, 5–14 years, 15–49 years, 50–69 years, and 70+ years) and sex and showed the proportion of CMNNDs, NCDs, and injuries to clarify sex- and age-specific patterns of the disease burden in North Korea. To identify health transitions during the March of Hardship in the 1990s, this study showed DALY rates with 95% uncertainty intervals (UI) at 5-year intervals between 1990 and 2019 by level 1 cause. We also compared the DALY rates by levels 2 and 3 causes to investigate which and how cause-specific DALY rates have changed over the past 30 years.

## Results

The death rates and DALY rates for all age groups decreased between 1990 and 2019 in North Korea and in all comparison groups. In North Korea, the age-standardized death rates and DALY rates decreased to 819.8 (95% UI 724.5–944.8) and 29442.0 (95% UI 25169.6–34426.6), in 2019, by 22.0% and 31.0%, respectively, compared to the 1990 values, with the largest reductions observed in children under 5 years (82.0% and 81.0%, respectively), and the least reductions observed in those aged 50–69 years (8.0% and 6.0%, respectively). Despite the large reduction in disease burden between 1990 and 2019, the percentage changes in age-standardized death rates and DALY rates were below the global level (-34.0% and -34.0%), and the levels for South Korea (-58.0% and -47.0%), and low-SDI countries (-37.0% and -41.0%), However, death rates were similar to that of former socialist countries (-21.0%) in the past, but DALY rates were higher in North Korea. In particular, the disease burden for children under 5 years in North Korea (DALY rates, 26504.9; 95% UI, 21665.5–32983.1) was lower than the global value (DALY rates, 71052.5; 95% UI, 60788.6–84146.5), and the value for low-SDI countries (DALY rates, 142891.3; 95% UI, 120210.4–171895.6), although the DALY rates among individuals aged 50–69 years (e.g., 50–69 years: DALY rates, 57192.0; 95% UI, 48544.3–65941.2) were higher than the global value (e.g., 50–69 years: DALY rates, 49080.5; 95% UI, 44131.2–54407.5) and the value for South Korea (e.g., 50–69 years: DALY rates 29805.2; 95% UI, 25747.6–34727.9) in 2019. Generally, the trends and patterns of age-standardized death rates and DALY rates were similar to those of former socialist countries (Table 1).

**Table 1:** Age-standardized and age-specific death rates and DALY rates in North Korea and four comparison nations between 1990 and 2019, and percentage change from 1990 to 2019.

Location	Global			North Korea			South Korea			Low-SDI Countries			Former socialist countries		
Age	1990	2019	change %	1990	2019	change %	1990	2019	change %	1990	2019	change %	1990	2019	change %
	Rate (95% UI)	Rate (95% UI)		Rate (95% UI)	Rate (95% UI)		Rate (95% UI)	Rate (95% UI)		Rate (95% UI)	Rate (95% UI)		Rate (95% UI)	Rate (95% UI)	
Death rates															
Age-standardized	1113.6 (1089.0–1137.7)	735.0 (699.0–771.0)	-34.0 (-37.3 to -30.6)	1053.7 (904.6–1219.4)	819.8 (724.5–944.8)	-22.2 (-33.9 to -8.9)	928.6 (923.8–933.4)	391.1 (378.3–404.6)	-57.9 (-59.3 to -56.3)	1799.2 (1725.1–1873.1)	1139.0 (1062.7–1226.5)	-36.7 (-40.8 to -32)	1017.2 (1012.0–1022.3)	799.7 (748.1–851.4)	-21.4 (-26.4 to -16.5)
<1 year	6174.1 (5866.7–6520.3)	2844.7 (2409.4–3393.7)	-53.9 (-60.8 to -44.9)	4962.0 (3907.4–6033.5)	1067.0 (844.8–1366.2)	-78.5 (-82.4 to -73.2)	1009.3 (870.0–1162.4)	245.1 (208.4–285.0)	-75.7 (-79.7 to -70.6)	10672.1 (10092.5–11318.6)	5101.9 (4238.4–6183.2)	-52.2 (-60.3 to -41.7)	2513.6 (2392.2–2646.8)	955.5 (814.5–1125.8)	-62.0 (-67.8 to -55.0)
Under 5	1895.4 (1793.7–2004.3)	760.9 (644.4–907.5)	-59.9 (-65.8 to -52.2)	1513.8 (1193.8–1837.8)	267.6 (211.9–341.2)	-82.3 (-85.5 to -78.0)	261.0 (227.1–297.1)	58.1 (50.5–66.7)	-77.8 (-81.1 to -73.5)	4087.7 (3866.6–4312.3)	1563.6 (1298.7–1895.1)	-61.7 (-68.2 to -53.6)	602.1 (569.7–639.5)	221.8 (189.3–261.4)	-63.2 (-68.8 to -56.6)
5–14 years	113.5 (109.8–117.7)	52.2 (47.6–57.5)	-54.0 (-57.9 to -49.9)	69.1 (52.2–99.2)	26.7 (23.2–31.2)	-61.4 (-71.2 to -51.3)	52.2 (49.3–55.3)	9.1 (8.7–9.5)	-82.6 (-83.8 to -81.3)	194.7 (183.7–205.4)	87.9 (77.4–100.5)	-54.8 (-60 to -48.7)	47.6 (46.7–48.6)	24.8 (23.2–26.7)	-48 (-51.3 to -44.1)
15–49 years	250.3 (242.0–259.0)	188.8 (177.3–200.7)	-24.5 (-29.3 to -19.7)	239.4 (174.0–314.3)	212.3 (152.9–302.5)	-11.3 (-41.7 to 34.1)	193.5 (192.7–194.4)	84.1 (80.6–87.7)	-56.6 (-58.4 to -54.7)	460.6 (428.1–499.4)	267.5 (240.5–298.5)	-41.9 (-47.4 to -35.6)	257.6 (256.7–258.4)	257.0 (237.6–277.3)	-0.2 (-7.8 to 7.6)
50–69 years	1621.8 (1568.8–1672.6)	1085.4 (1020.4–1147.3)	-33.1 (-37.3 to -28.7)	1530.3 (1207.4–1915.5)	1410.7 (1171.4–1632)	-7.8 (-29.2 to 16.1)	1403.3 (1397.1–1409.6)	492.8 (471.3–515.3)	-64.9 (-66.4 to -63.3)	2610.7 (2462.3–2765.3)	1732.5 (1580.6–1890.4)	-33.6 (-39.4 to -27.8)	1718.4 (1712.3–1724.3)	1448.8 (1336.5–1561.4)	-15.7 (-22.1 to -9.1)
70+ years	7708.7 (7573.7–7832.7)	6126.7 (5900.4–6329.8)	-20.5 (-23.6 to -17.5)	7617.6 (6847.9–8416.8)	6787.6 (6301.4–7437.0)	-10.9 (-19.6 to -0.2)	7351.8 (7327.3–7377.1)	4187.3 (4058.1–4322.2)	-43 (-44.8 to -41.1)	9791.0 (9485.6–10085.3)	7789.1 (7452.2–8127.1)	-20.4 (-24.1 to -16.6)	8343.7 (8324.5–8365.6)	7290.9 (6891.9–7701.3)	-12.6 (-17.4 to -7.7)
DALY rates															
Age-standardized	50059.9 (46999.2–53544.6)	32857.0 (29648.3–36403.3)	-34.4 (-38.2 to -30.5)	42501.6 (36973.4–49307.0)	29442.0 (25169.6–34426.6)	-30.7 (-39.4 to -21.4)	32256.4 (29591.7–35278.4)	17191.6 (14738.5–19958.2)	-46.7 (-50.3 to -43.2)	84125.4 (79317.0–89064.2)	49330.8 (44266.2–54849.7)	-41.4 (-45.9 to -36.3)	38546.1 (35708.9–41693.2)	30254.8 (27204.6–33652.6)	-21.5 (-25.6 to -17.4)
<1 year	551850.9 (524624.7–582910.5)	256548.4 (217805.3–305257.2)	-53.5 (-60.3 to -44.5)	443298.4 (350017.5–539278.9)	97624.9 (78488.7–124446.5)	-78.0 (-81.9 to -72.7)	91855.4 (79402.6–105473.1)	23675.7 (20369.8–27276.0)	-74.2 (-78.5 to -69.2)	952480.4 (901347.2–1009239.8)	458355.8 (382163.3–553659.1)	-51.9 (-59.9 to -41.4)	225987.0 (215246.4–237958.4)	87418.4 (74687.5–102578.7)	-61.3 (-67 to -54.4)
Under 5	170928.2 (162091.9–181113.9)	71052.5 (60788.6–84146.5)	-58.4 (-64.3 to -50.9)	136902.5 (108612.3–166099.2)	26504.9 (21665.5–32983.1)	-80.6 (-84.0 to -76.2)	7797.9 (6715.0–9025.8)	26041.9 (23039.3–29460.0)	-70.1 (-74.4 to -64.9)	364385.5 (345456.2–385082.2)	142891.3 (120210.4–171895.6)	-60.8 (-67.0 to -52.6)	56415.8 (53468.4–59759.1)	22714.1 (19677.1–26341.7)	-59.7 (-65.0 to -53.4)
5–14 years	14129.3 (12627.1–15935.1)	9065.7 (7569.1–10733.9)	-35.8 (-40.3 to -31.6)	9417.7 (7392.7–12240.8)	5931.5 (4784.5–7287.8)	-37 (-49.8 to -25.6)	8300.3 (7057.6–9755.8)	4505.8 (3426.7–5796.0)	-45.7 (-52.2 to -39.4)	22303.3 (20151.7–24793.6)	12977.1 (10987.8–15305.5)	-41.8 (-46.9 to -36.9)	7901.2 (6721.9–9315)	5885.9 (4771.0–7163.0)	-25.5 (-29.6 to -21.8)
15–49 years	23701.5 (20961.8–26815.0)	20016.1 (17238.6–22979.6)	-15.5 (-18.9 to -12.4)	20841.2 (16590.1–25721.7)	19162.4 (15370.1–24239.2)	-8.1 (-28.7 to 18.2)	19771.6 (17235.3–22634.0)	13643.3 (11099.5–16559.3)	-31.0 (-35.6 to -27.0)	36767.2 (33331.3–40756.5)	24808.7 (21742.7–28190.1)	-32.5 (-37.3 to -28.1)	23628.5 (20930.0–26719.6)	23049.3 (20246.5–26406.0)	-2.5 (-6.5 to 1.7)
50–69 years	65188.3 (60302.2–70772.4)	49080.5 (44131.2–54407.5)	-24.7 (-28.5 to -21.4)	60973.6 (50035.4–73478.7)	57192.0 (48544.3–65941.2)	-6.2 (-24.2 to 11.9)	58274.8 (53948.4–63445.2)	29805.2 (25747.6–34727.9)	-48.9 (-52.6 to -45.1)	96623.9 (89983.5–104262.1)	69841.2 (62928.3–77352.9)	-27.7 (-33.0 to -22.5)	68810.2 (63970.5–74369.9)	59492.8 (53649.8–66251.0)	-13.5 (-18.3 to -8.8)
70+ years	132473.8 (125331.1–140202.1)	104606.1 (97413.4–112097.0)	-21.0 (-23.8 to -18.4)	132199.4 (119768.2–145113.1)	117751.0 (108181.7–128628.9)	-10.9 (-18.3 to -3)	129165.4 (122679.5–136199.9)	74713.6 (68282.8–81757.7)	-42.2 (-44.7 to -39.8)	174462.2 (166445.8–183527.6)	137131.7 (128463.5–146326.8)	-21.4 (-24.7 to -18.1)	137289.0 (130512.7–144888.5)	116270.3 (108256.0–125781.2)	-15.3 (-19.1 to -11.5)

Data in parentheses are 95% uncertainty intervals. DALY = disability-adjusted life-years. For Low-SDI countries and Central Europe, Eastern Europe, and Central Asia, the average rates have been reported.

Table 2 shows that age-standardized death rates and DALY rates for all level 1 causes decreased in North Korea and the four comparison groups. The decrease in age-standardized death rate was largest in CMNNDs for North Korea (percentage change, -62.0%) and smallest in NCDs for North Korea (percentage change, -14.0%). In comparison to the DALY rates for CMNNDs for North Korea (DALY rates, 3443.9; 95% UI, 2865.9–4275.9), the rate for CMNNDs for low-SDI countries was 6.3 times higher (DALY rates, 21971.7; 95% UI, 19205.7–25281.9) and the global value was 2.7 times higher (DALY rates, 9483.4; 95% UI, 8394.5–10801.7), but almost equal to those for former socialist countries (DALY rates, 3252.3; 95% UI, 2898.2–3659.6). However, the DALY rates of NCDs for North Korea were similar to the global value (DALY rates, 20204.9; 95% UI, 17826.0–22636.8), low-SDI countries (DALY rates, 23340.0; 95% UI 20727.0–26140.4), and former socialist countries (DALY rates, 22834.1; 95% UI, 20524.7–25498.0). Age-standardized death rates and DALY rates for injuries in North Korea decreased by more than 25%, which was similar to the trends globally, and in low-SDI countries, as well as in former socialist countries. The proportion of death and DALY rates from NCDs is higher than CMNNDs in North Korea and global, South Korea, and former socialist countries (S1 Fig).

**Table 2:** Age-standardized death rates and DALY rates in North Korea and four comparison nations between 1990–2019, and percentage change from 1990 to 2019, by level 1 cause.

Location	Global			North Korea			South Korea			Low-SDI Countries			Former socialist countries		
	1990	2019	change %	1990	2019	change %	1990	2019	change %	1990	2019	change %	1990	2019	change %
	Rate (95% UI)	Rate (95% UI)		Rate (95% UI)	Rate (95% UI)		Rate (95% UI)	Rate (95% UI)		Rate (95% UI)	Rate (95% UI)		Rate (95% UI)	Rate (95% UI)	
Death rates															
CMNNDs	298.3 (281.8–315.0)	140.7 (126.8–157.9)	-52.8 (-57.4 to -47.4)	157.9 (132.3–186.9)	60.2 (49.5–74.9)	-13.9 (-27.3 to 1.2)	69.5 (64.4–77.6)	29.5 (21.2–33.3)	-63 (-68.2 to -59.1)	920.4 (845.2–992.9)	409.5 (363.5–465.2)	-56.9 (-61.6 to -50.9)	72.2 (69.1–75.4)	43.0 (39.4–47.1)	-40.4 (-45.8 to -34.4)
NCDs	730.6 (710.4–751.0)	539.6 (515.1–563.4)	-26.1 (-29.7 to -22.6)	806.5 (690.5–933.8)	694.2 (619.2–794.5)	-61.9 (-67.8 to -53.8)	774.7 (754.5–782.6)	319.0 (306.5–334.4)	-43.6 (-47.9 to -39.4)	765.9 (693.7–835.5)	650.7 (600.5–701.3)	-14 (-19.9 to -7.3)	694.1 (650.1–737.6)	850.6 (847.5–853.7)	-33.7 (-38.9 to -28.3)
Injuries	84.7 (80.6–88.3)	54.7 (49.8–58.7)	-35.5 (-40.1 to -30.4)	89.3 (70.4–116.0)	65.4 (50.7–85.6)	-26.7 (-44.9 to -3.7)	84.4 (80.6–98.7)	42.6 (35.9–46.1)	-50.8 (-57.8 to -46.6)	112.9 (101.8–123.2)	78.8 (69.5–87.8)	-33.5 (-39.6 to -25.9)	94.4 (93.2–95.7)	62.6 (57.3–67.8)	-18.4 (-23.5 to -13.4)
DALY rates															
CMNNDs	19962.1 (18775.3–21220.3)	9483.4 (8394.5–10801.7)	-52.5 (-57.7 to -46.1)	11109.5 (8954.9–13534.8)	3443.9 (2865.9–4275.9)	-69.0 (-74.2 to -62.4)	3292.6 (2928.1–3730.6)	1217.1 (1039.7–1426.2)	-57.6 (-72.5 to -52.1)	50938.6 (47620.8–54275.8)	21971.7 (19205.7–25281.9)	-55.5 (-60.2 to -49.6)	5927.4 (5585.7–6336.7)	3252.3 (2898.2–3659.6)	-45.1 (-50.6 to -39.2)
NCDs	25054.0 (22560.9–27543.2)	20204.9 (17826.0–22636.8)	-19.4 (-23.0 to -15.6)	26227.5 (22487.3–30604.7)	22644.7 (19402.8–26270.3)	-13.7 (-25.9 to -0.7)	24003.0 (21867.8–26356.1)	13534.6 (11432.6–15827.8)	-49.5 (-62.1 to -44.2)	27141.1 (24205.4–30118.8)	23340.0 (20727.0–26140.4)	-30.2 (-36.7 to -22.2)	26386.7 (24250.7–28750.8)	22834.1 (20524.7–25498)	-13.5 (-17.5 to -9.4)
Injuries	5043.8 (4694.3–5423.4)	3168.7 (2882.5–3493.5)	-37.2 (-41.1 to -32.8)	5164.6 (4063.5–6563)	3353.4 (2660.4–4346.8)	-35.1 (-49.6 to -15.6)	4960.9 (4559.4–5447.4)	2439.9 (2101.9–2844.2)	-58.8 (-60.5 to -55.9)	6045.7 (5454.4–6648.4)	4019.0 (3532.5–4549.3)	-15 (-22.3 to -6.9)	6232 (5737.8–6843)	4168.5 (3712.6–4748.9)	-33.1 (-37 to -29.1)

Data in parentheses are 95% uncertainty intervals. DALY = disability-adjusted life-years. CMNNDs = communicable, maternal, neonatal, and nutritional diseases. NCDs = non-communicable diseases. For Low-SDI countries and Central Europe, Eastern Europe, and Central Asia, the average rates have been reported.

**Fig 1:**
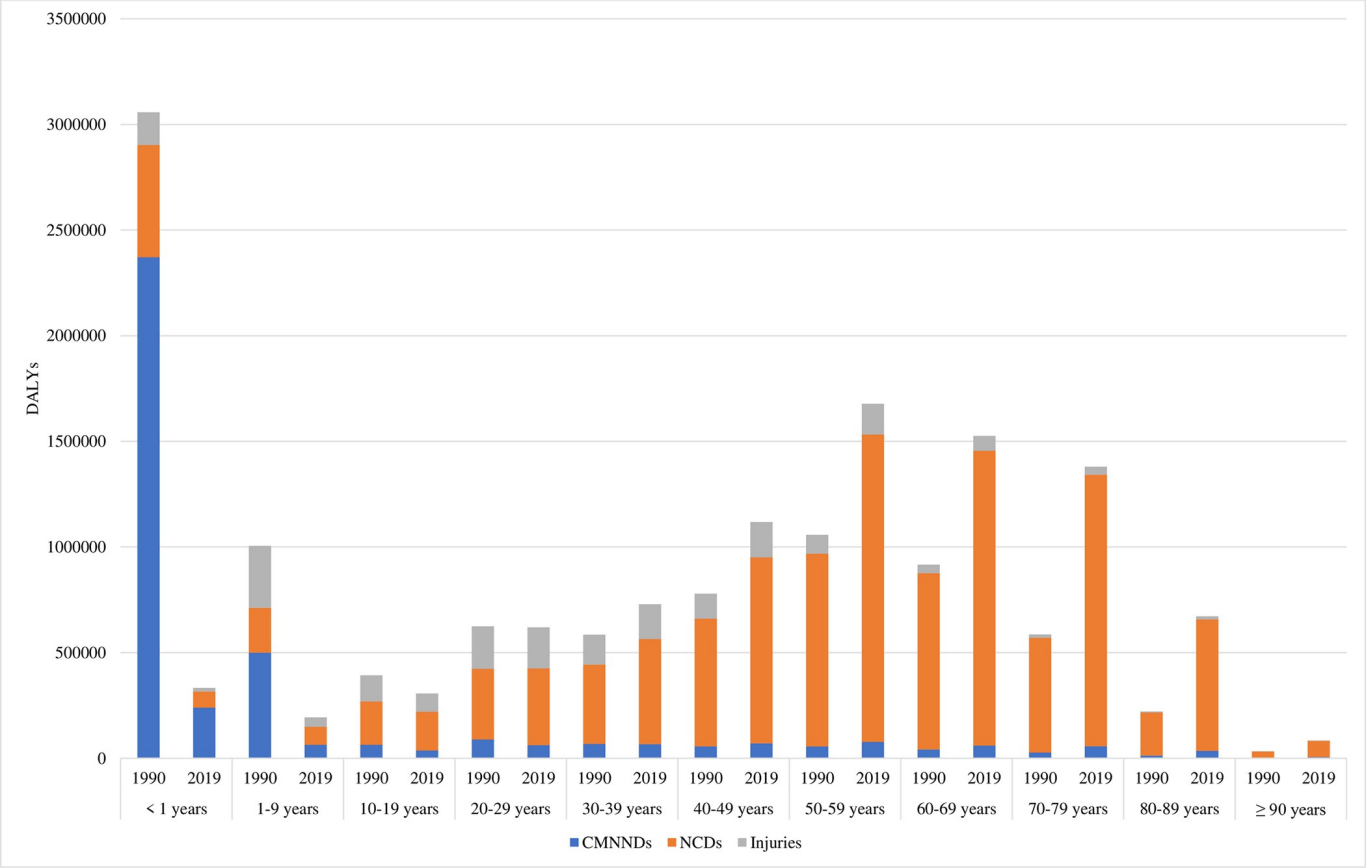
DALYs for communicable, maternal, neonatal, and nutritional diseases (CMNNDs), non-communicable diseases (NCDs), and injuries in North Korea by age, 1990 to 2019. DALY = disability-adjusted life-years.

Fig 1 presents the age-specific DALYs and their proportions caused by CMNNDs, NCDs, and injuries in North Korea in 1990 and 2019. Generally, the DALYs decreased for those under 20 years, whereas the DALYs for adults in their 30s and older increased significantly over time. From those in their 30s, the burden of disease caused by NCDs has steadily increased over the past 30 years, with those in their 60s increasing by more than 67% in 2019 and those in their 70s by more than 136.8% compared to the 1990 values. Although the DALYs of children under one year of age were the highest due to the largest proportion of DALYs for CMNNDs in 1990, the largest proportional cause-specific changes in DALYs under one year of age between 1990 and 2019 were for CMNNDs, from 2371402.0 DALYs in 1990 to 240159.9 DALYs in 2019. In addition, CMNNDs 36810.0 and injuries 86201.1 in 2019 decreased compared to 1990. On the other hand, the DALYs peaked in individuals aged 50–59 years, followed by the highest DALYs in those aged 60–69 years, and then in those aged 70–79 years in 2019. Among these age groups, the largest proportion of DALYs was largely caused by NCDs.

**Fig 2:**
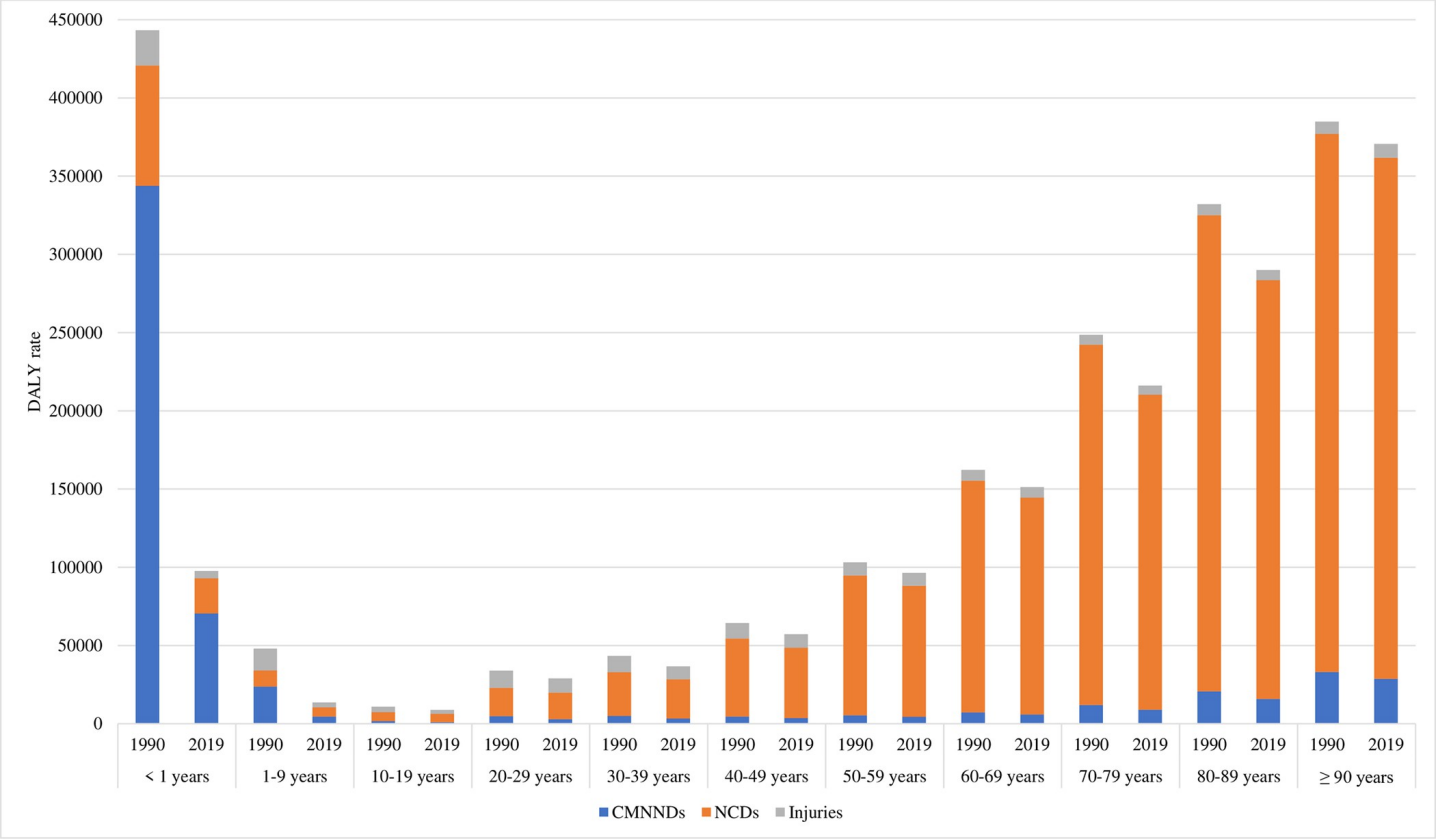
DALY rate for communicable, maternal, neonatal, and nutritional diseases (CMNNDs), non-communicable diseases (NCDs), and injuries in North Korea by age, 1990 to 2019. DALY = disability-adjusted life-years.

Fig 2 shows the age-specific DALY rates caused by North Korea’s CMNNDs, NCDs, and injuries from 1990 to 2019. It shows a decreasing burden of disease over time. Although the highest reduction of DALY rates was among the under 1 year age group, they were still exposed to the high disease burden of infectious diseases. On the other hand, the disease burden of NCDs decreased but remained high among adult groups. The proportion of DALYs by age group was similar for both male and female, but the age distribution of DALYs by sex was slightly different in 2019 (S2 Fig).

**Fig 3:**
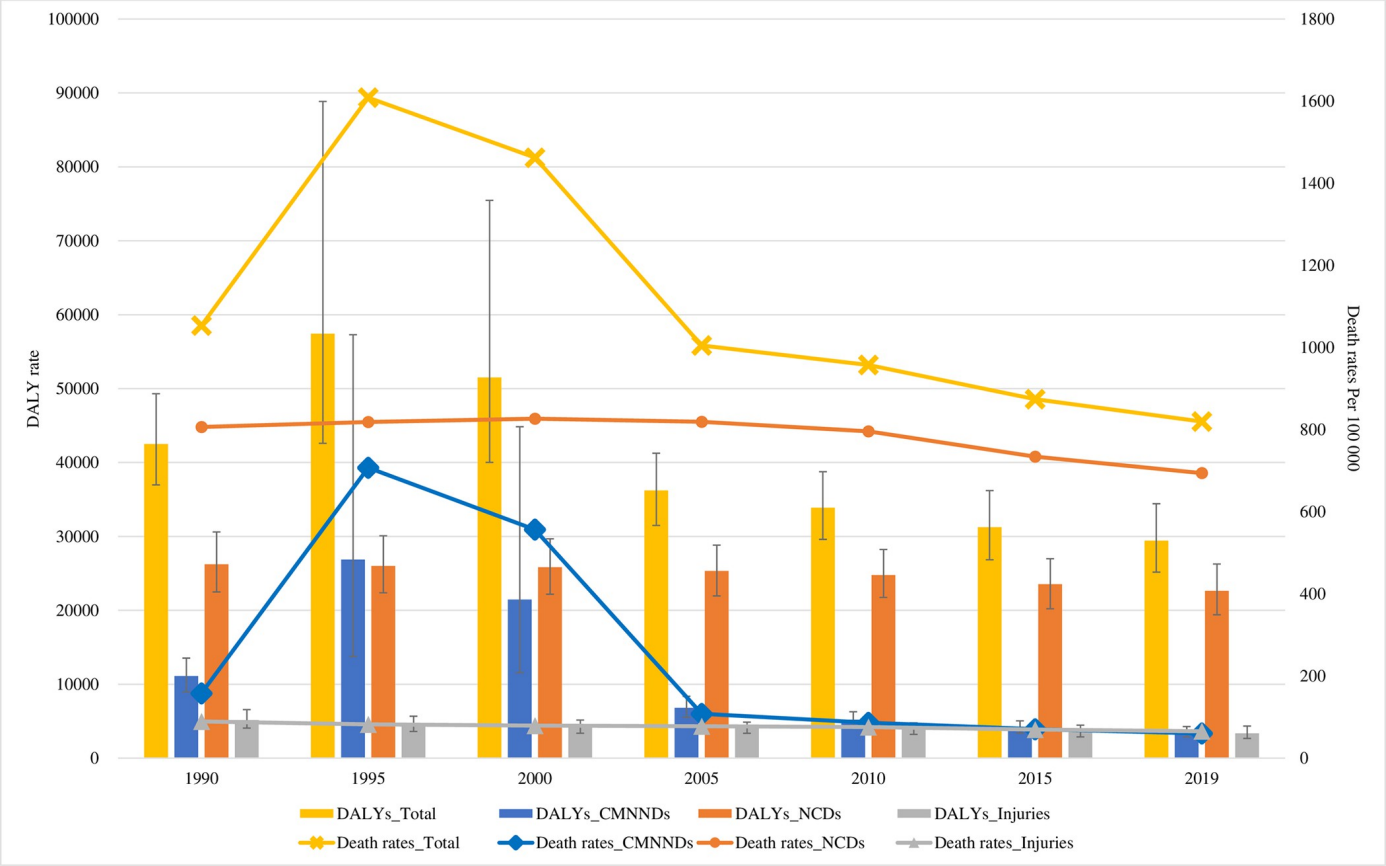
Trends in age-standardized deaths and DALY rate by communicable, maternal, neonatal, and nutritional diseases (CMNNDs), non-communicable diseases (NCDs), and injuries, 1990 to 2019. DALY = disability-adjusted life-years.

Between 1990 and 2019, the DALY rates caused by CMNNDs sharply peaked in 1995 (DALY rates, 26865.5; 95% UI, 13575.4–57287.1) and then showed a steep decreasing trend after 2000 (DALY rates, 21451.2; 95% UI, 44835.0–11558.8). The DALY rates for NCDs and injuries decreased from 26227.5 (95% UI, 22487.3–30604.7) and 5164.6 (95% UI, 4063.5–6563.0) in 1990 to 22644.7 (95% UI, 19402.8–2670.3) and 3353.0 (95% UI, 2660.0–4346.8), respectively in 2019. However, infectious diseases changed, while NCDs and injuries changes were insignificant. These trends and patterns were similar to the death rates (Fig 3).

**Fig 4:**
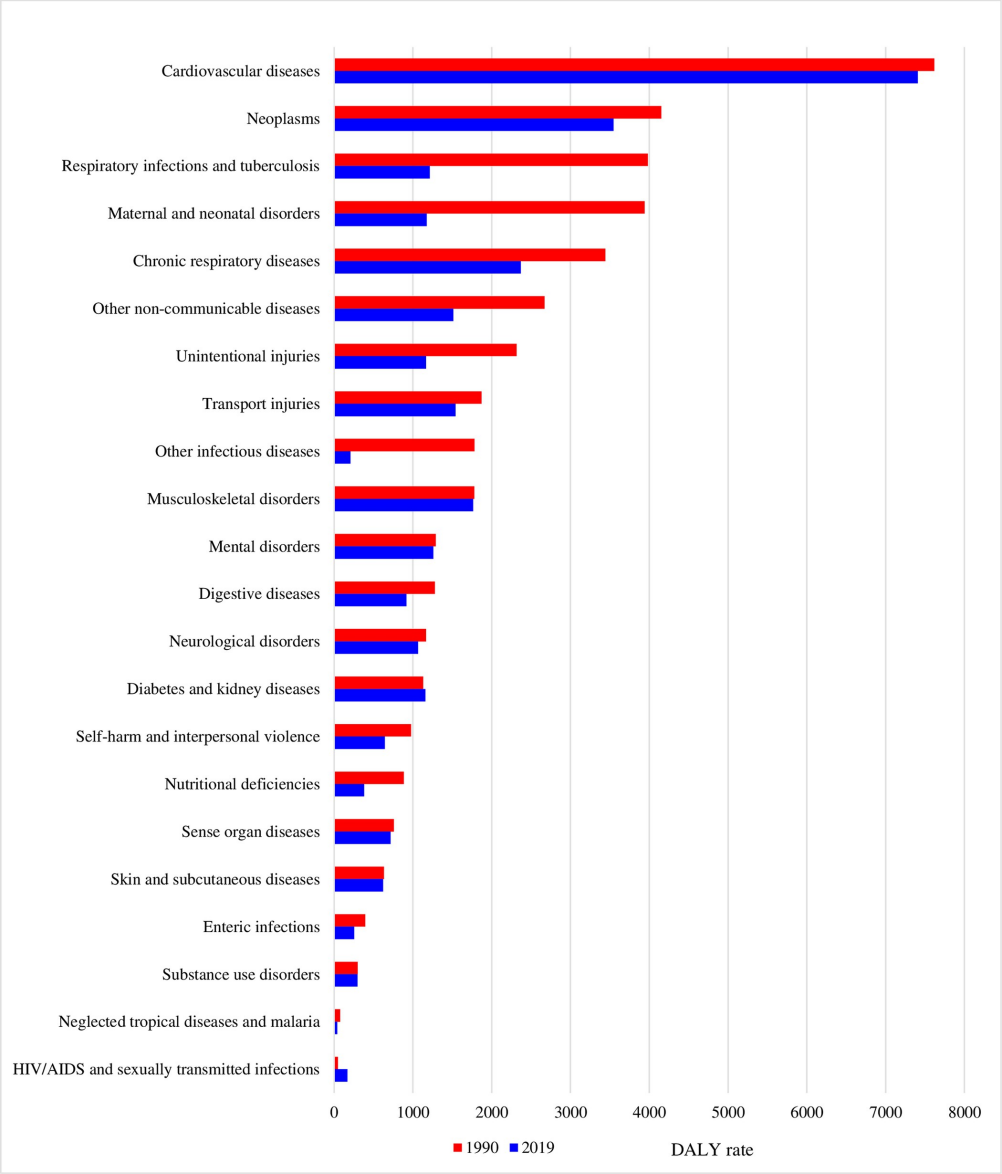
Age-standardized DALY rate for every level 2 cause, 1990 to 2019. DALY = disability-adjusted life-years.

Fig 4 shows age-standardized DALY rates for all level 2 causes in 1990 and 2019. The DALY rates for all level 2 causes decreased, except HIV/AIDS, sexually transmitted infections, diabetes, and kidney diseases. Cardiovascular diseases and neoplasms were the first and second causes of age-standardized DALY rates in 1990, and these ranks were maintained in 2019 (cardiovascular diseases: DALY rates, 7410.7, 95% UI, 6281.3–8713.1; Neoplasms: DALY rates, 3546.2, 95% UI, 2850.1–4344.0). In particular, stroke and ischemic heart disease are the highest disease burden and premature deaths in level 3 (S3 Fig). During this period, chronic respiratory diseases and musculoskeletal disorders surpassed respiratory infection and tuberculosis and maternal and neonatal disorders to become the third- and fourth-leading causes of age-standardized DALY rates. The largest decline in the rates of age-standardized DALY rates for level 2 causes occurred for respiratory infection and tuberculosis, maternal and neonatal disorders, and other infectious diseases (S1 Table).

## Discussion

This study comprehensively described the burden of disease in North Korea. To our knowledge, this is the first study to analyze the trends and patterns of the disease burden in North Korea from 1990 to 2019. This study also compared the disease burden in North Korea with that of other countries, including low-SDI countries, South Korea, and former socialist countries. Worldwide, child death rates have declined by more than 50% over the past 30 years. In particular, North Korea’s child death rates rate is more than twice as high as the rate globally and in low-SDI countries. Death rates among adults had not decreased and most of the differences in the disease burden were largely due to NCDs. Although North Korea reduced its death rates and DALY rates by 22.2% and 30.7%, respectively, in comparison with the rate in 1990, these reductions were lower than the average rate globally, in low-SDI countries and in South Korea. The death rates and DALY rates for children under 5 years dramatically decreased by more than 80%, largely due to a sharp decline in CMNNDs, similar to trends in South Korea, whereas the decline in death rates and DALY rates for adults was less than the average globally, in low-SDI countries, and in South Korea. In particular, the burden of disease remains high for those aged ≥50 years. The high burden of diseases is largely due to the persistently high burden of NCDs. Our findings indicate that cardiovascular diseases, neoplasms, and chronic respiratory diseases were the leading causes of disease in 1990 and 2019.

The disease burden in North Korea differs from that in low-income countries in terms of background and patterns. Compared to the results for low-SDI countries and the global average, the child death rates had surprisingly decreased, but the death rates among adults had not much decreased and most of the differences were largely due to NCDs. These trends and patterns of the disease burden in North Korea were similar to those in former socialist countries. This phenomenon appears to be because North Korea and the former socialist countries had very similar healthcare systems and population health policies and shared their achievements and limitations [14]. Considering the disease burden in North Korea, former socialist countries may, similarly, have NCDs as their main burden of disease. However, controlling infectious diseases remains a top priority in public health. Additionally, international medical support for North Korea has been centered on infectious diseases and malnutrition because children and patients with tuberculosis are considered the most vulnerable groups, indicating that national management of NCDs has poor performance [15, 16]. Therefore, death rates and DALY rates have decreased at a slower rate than in comparative groups, suggesting that adult health has become relatively more important in North Korea due to poor quality of life, poor health conditions, and adult poverty.

Communicable diseases, which were effectively managed by the health systems before 1990, deteriorated in the 1990s. One of the key aspects of the health transition in North Korea is the impact of economic difficulties. During the 1990s, North Korea experienced the March of Hardship, which resulted in an estimated one million deaths due to starvation [17]. Higher death rates and DALY rates were observed among children than among adults during these periods, with the most common CMNNDs as the cause of death including malaria, diarrheal diseases, measles, respiratory infection, tuberculosis, and maternal and neonatal disorders [18]. The North Korean government called for food assistance, and WFP and other donor nations urgently responded to their requests by providing food and medical supplies to the vulnerable populations such as children, pregnant women, and malnourished people [18, 19]. Through international assistance and communicable disease-oriented health systems, the death rates and DALY rates from CMNNDs dramatically decreased after the March of Hardship.

Unlike developed countries such as South Korea, which drastically reduced the death rates due to NCDs, in North Korea, the proportion of deaths and DALY rates from NCDs continues to increase (S1 Fig). Moreover, most of the disease burden and premature deaths were from chronic diseases, including cardiovascular diseases such as stroke and ischemic heart disease (S3 Fig). Poverty does not directly cause cardiovascular disease or neoplasm; however, in low-income countries, the focus is generally on economic development and infectious disease management; thus, there is inadequate national interest and resources directed toward chronic disease management [20]. These findings indicate that although people in poorer countries manage their health when they experience economic difficulties, despite living longer, many suffer from chronic diseases due to poor living standards and quality of life or die relatively early.

Our finding demonstrated that DALY rates decreased after the 1-year age group to 10 years age group, but the rates increased remarkably from 20 years to the last age group in both 1990 and 2019 mainly due to NCDs.

Based on the thrift phenotype hypothesis, groups of people who are malnourished in their infancy or developmental phase are more susceptible to the development of NCDs as they age [21]. Several studies have demonstrated the long-term effects of famine on the chronic disease burden [22–27]. A systematic review of the impact of the “Great Leap Forward Famine” on long-term health conditions reported that the experience of famine might increase the risk of chronic conditions later in life among individuals in China. This suggests that the adverse impact of famine would be more evident as the exposed individuals grow older [28]. In particular, several studies have shown that early-life exposure to the Chinese famine might be associated with an increased cardiovascular risk in adulthood [22, 23, 27] because such exposure impairs glucose metabolism, thereby increasing the risk of dysfunction in the metabolic syndrome, and deleterious impacts from cardiovascular diseases [29]. Likewise, North Korean defectors who are stunted due to severe malnutrition are more likely to experience a metabolic adaptation that favors fat deposition [30]. This results in an increased risk of obesity and metabolic syndrome after transitioning to a westernized lifestyle and dietary changes [31]. Therefore, chronic economic difficulties combined with increasing age would continue to place a greater burden on non-infectious diseases among the North Korean population. A majority of the North Korean population die from NCDs, and the older adult population aged 65 and over accounts for 10% of the population. For the North Korean authorities to effectively manage the health of the North Korean population, a transition to a new paradigm of population lifestyle management and health promotion is needed. In addition, the international community must continuously persuade the North Korean authorities that investment in NCDs is a wise economic investment, provide specific economical interventions applicable to low income countries such as North Korea, and systematically provide technical, material, and human support for such investments.

In 2015, the international community signed on to achieve the UN’s Sustainable Development Goal (SDG) target 3.4, which aims to reduce premature deaths from four major NCDs, particularly among those between the ages of 30 and 70, by one-third, by 2030. Given the current trend, it will be difficult for North Korea to achieve this [32], indicating that the service delivery for chronic disease in healthcare systems might be inadequate due to underfunding and an orientation toward CMNNDs. The prevention and management of chronic diseases may not be sufficiently implemented without the support of the global health community. Given the lack of healthcare infrastructure and resources, external support and collective efforts from international agencies, bilateral, and civil society organizations are needed to accelerate progress toward reducing the burden of NCDs. Global health community assistance strategies should consider the transition to longer-term strategies in chronic disease sectors.

This study has several limitations. Broadly, the general methodological limitations of the GBD data have been published elsewhere [11]. Although this study provided a comprehensive disease burden based on the estimates from the GBD study, a major limitation is that the data from the GBD study, especially for low-income countries, including North Korea, is influenced by the quality of the primary data [33]. Since large uncertainties surround the estimates of disease burden in North Korea, the interpretation of comparisons within or between countries should always be performed with caution. Another limitation is that this study did not analyze regional differences in the burden of disease because the GBD study does not report estimates by regions or subpopulations in North Korea. Since malnutrition rates, such as rates of stunted and underweight children were highest in the northeast area of North Korea, the regional variations of the disease burden in North Korea are important [5]. Further studies are warranted to examine whether the disease burden differs by region.

The strength of this study is a comprehensive study that analyzes the trends and patterns of disease burden in North Korea, analyzed the North Korean disease burden compared to four groups, and also investigated changes in disease burden by disease category and age group between 1990 and 2019.

NCDs cannot be completely cured and gradually worsen, leading to a great loss of labor and productivity over a lifetime and increasing the economic burden of poor health. The North Korean population has one of the highest probabilities of dying from NCDs. Considering the chronic food shortage and failure of the medical system in North Korea, the burden of chronic diseases is expected to increase further in the future. To effectively manage the health of the North Korean population, expansion of essential medical services, along with a new paradigm, are essential. Thus, NCDs should no longer be neglected but should be prioritized in public health agendas in North Korea.

## Supporting information

S1 Fig Proportion of age-standardized DALY rates by level 1 cause in North Korea and four comparison nations, 1990 to 2019. DALY = disability-adjusted life-year. CMNN = communicable, maternal, neonatal, and nutritional. NCDs = non-communicable diseases.
(DOCX)

S2 Fig DALYs for communicable, maternal, neonatal, and nutritional disease (CMNNDs), non-communicable diseases (NCDs), and injuries in North Korea by age and sex, 1990 to 2019. DALY = disability-adjusted life-years.
(DOCX)

S3 Fig Age-standardized DALY rates for 30 leading causes of level 3 in both sexes in 2019. DALY = disability-adjusted life-years. YLLs = years of life lost. YLDs = years of life lived with disability.
(DOCX)

S1 Table Age-standardized DALY rates for every level 2 cause, 1990 to 2019. (DOCX)

S1 File Inclusivity in global research. (DOCX)
