# Correction: Trends and patterns of North Korea’s disease burden from 1990 to 2019: Results from Global Burden of Disease study 2019

**DOI:** 10.1371/journal.pone.0289257

**Published:** 2023-07-24

**Authors:** Eun Hae Lee, Minjae Choi, Joshua Kirabo Sempungu, Yo Han Lee

There are errors in the Funding statement. The correct Funding statement is as follows: YHL was supported by grants from the National Research Foundation (NRF) of Korea funded by the Ministry of Science and ICT [grant number: NRF-2021R1G1A1095517] as well as from Korea University. The funders had no role in study design, data collection and analysis, decision to publish, or preparation of the manuscript.

There is an error in Table 2. The values of Communicable-malnutrition-maternal-newborn diseases (CMNND) and Non-communicable diseases are swapped. Please see the correct Table 2 here.

**Table 2:** Age-standardized death rates and DALY rates in North Korea and four comparison nations between 1990–2019, and percentage change from 1990 to 2019, by level 1 cause.

Location	Global			North Korea			South Korea			Low-SDI Countries			Former socialist countries		
Age	1990	2019	change %	1990	2019	change %	1990	2019	change %	1990	2019	change %	1990	2019	change %
	Rate (95% UI)	Rate (95% UI)		Rate (95% UI)	Rate (95% UI)		Rate (95% UI)	Rate (95% UI)		Rate (95% UI)	Rate (95% UI)		Rate (95% UI)	Rate (95% UI)	
Death rates															
CMNNDs	298.3 (281.8 to 315.0)	140.7 (126.8 to 157.9)	-52.8 (-57.4 to -47.4)	157.9 (132.3 to 186.9)	60.2 (49.5 to 74.9)	-61.9 (-67.8 to -53.8)	69.5 (64.4 to 77.6)	29.5 (21.2 to 33.3)	-57.6 (-72.5 to -52.1)	920.4 (845.2 to 992.9)	409.5 (363.5 to 465.2)	-61.9 (-67.8 to -53.8)	72.2 (69.1 to 75.4)	43.0 (39.4 to 47.1)	-40.4 (-45.8 to -34.4)
NCDs	730.6 (710.4 to 751.0)	539.6 (515.1 to 563.4)	-26.1 (-29.7 to -22.6)	806.5 (690.5 to 933.8)	694.2 (619.2 to 794.5)	-13.9 (-27.3 to 1.2)	774.7 (754.5 to 782.6)	319.0 (306.5 to 334.4)	-58.8 (-60.5 to -55.9)	765.9 (693.7 to 835.5)	650.7 (600.5 to 701.3)	-13.9 (-27.3 to 1.2)	850.6 (847.5 to 853.7)	694.1 (650.1 to 737.6)	-18.4 (-23.5 to -13.4)
Injuries	84.7 (80.6 to 88.3)	54.7 (49.8 to 58.7)	-35.5 (-40.1 to -30.4)	89.3 (70.4 to 116.0)	65.4 (50.7 to 85.6)	-26.7 (-44.9 to -3.7)	84.4 (80.6 to 98.7)	42.6 (35.9 to 46.1)	-49.5 (-62.1 to -44.2)	112.9 (101.8 to 123.2)	78.8 (69.5 to 87.8)	-26.7 (-44.9 to -3.7)	94.4 (93.2 to 95.7)	62.6 (57.3 to 67.8)	-33.7 (-38.9 to -28.3)
DALY rates															
CMNNDs	19962.1 (18775.3 to 21220.3)	9483.4 (8394.5 to 10801.7)	-52.5 (-57.7 to 46.1)	11109.5 (8954.9 to 13534.8)	3443.9 (2865.9 to 4275.9)	-69.0 (-74.2 to -62.4)	3292.6 (2928.1 to 3730.6)	1217.1 (1039.7 to 1426.2)	-63.0 (-68.2 to -59.1)	50935.6 (47620.8 to 54275.8)	21971.7 (19205.7 to 25281.9)	-56.9 (-61.6 to -50.9)	5927.4 (5585.7 to 6336.7)	3252.3 (2898.2 to 3659.6)	-45.1 (-50.6 to -39.2)
NCDs	25054.0 (22560.9 to 27543.2)	20204.9 (17826.0 to 22636.8)	-19.4 (-23.0 to -15.6)	26227.5 (22487.3 to 30604.7)	22644.7 (19402.8 to 26270.3_	-13.7 (-25.9 to -0.7)	24003.0 (21867.8 to 26356.1)	13534.6 (11432.6 to 15827.8)	-43.6 (-47.9 to -39.4)	27141.1 (24205.4 to 30118.8)	23340.0 (20727.0 to 26140.4)	-14.0 (-19.9 to -7.3)	26386.7 (24250.7 to 28750.8)	22834.1 (20524.7 to 25498.0)	-13.5 (-17.5 to -9.4)
Injuries	5043.8 (4694.3 to 5423.4)	3168.7 (2882.5 to 3493.5)	-37.2 (-41.1 to -32.8)	5164.6 (4063.5 to 6563.0)	3353.4 (2660.4 to 4346.8)	-35.1 (-49.6 to -15.6)	4960.9 (4559.4 to 5447.4)	2439.9 (2101.9 to 2844.2)	-50.8 (-57.8 to -46.6)	6045.7 (5454.4 to 6648.4)	4019.0 (3532.5 to 4549.3)	-33.5 (-39.6 to -25.9)	6232.0 (5737.8 to 6843.0)	4168.5 (3712.6 to 4748.9)	-33.1 (-37.0 to -29.1)

Data in parentheses are 95% uncertainty intervals. DALY = disability-adjusted life-years. CMNNDs = communicable, maternal, neonatal, and nutritional diseases. NCDs = non-communicable diseases. For Low-SDI countries and Central Europe, Eastern Europe, and Central Asia, the average rates have been reported.
