# Supplementary material for: Trends and patterns of North Korea’s disease burden from 1990 to 2019: Results from Global Burden of Disease study 2019
Source: PLoS One. 2022 Nov 14;17(11):e0277335. doi: 10.1371/journal.pone.0277335 (PMC9662722; doi:10.1371/journal.pone.0277335)
Supplement: S1 Fig — DALY = disability-adjusted life-year. CMNN = communicable, maternal, neonatal, and nutritional. NCDs = non-communicable diseases. (DOCX) [file pone.0277335.s001.docx]

[Supporting information]

Supporting information 1 Fig. Proportion of age-standardized DALY rates by level 1 cause in North Korea and four comparison nations, 1990 to 2019.
DALY=disability-adjusted life-year. CMNN=communicable, maternal, neonatal, and nutritional. NCDs=non-communicable diseases
