# Supplementary material for: Trends and patterns of North Korea’s disease burden from 1990 to 2019: Results from Global Burden of Disease study 2019
Source: PLoS One. 2022 Nov 14;17(11):e0277335. doi: 10.1371/journal.pone.0277335 (PMC9662722; doi:10.1371/journal.pone.0277335)
Supplement: S2 Fig — DALY = disability-adjusted life-years. (DOCX) [file pone.0277335.s002.docx]

Supporting information 2 Fig. DALYs for communicable, maternal, neonatal, and nutritional disease (CMNND), non-communicable diseases (NCDs), and injuries in North Korea by age and sex, 1990 to 2019.
DALY=disability-adjusted life-years.

Male

Female
