# Supplementary material for: Trends and patterns of North Korea’s disease burden from 1990 to 2019: Results from Global Burden of Disease study 2019
Source: PLoS One. 2022 Nov 14;17(11):e0277335. doi: 10.1371/journal.pone.0277335 (PMC9662722; doi:10.1371/journal.pone.0277335)
Supplement: S3 Fig — DALY = disability-adjusted life-years. YLLs = years of life lost. YLDs = years of life lived with disability. (DOCX) [file pone.0277335.s003.docx]

Supporting information 3 Fig. Age-standardized DALY rate for 30 leading causes of level 3 in both sexes in 2019.
DALY=disability-adjusted life-years. YLLs=years of life lost. YLDs=years of life lived with disability
