# Supplementary material for: Trends and patterns of North Korea’s disease burden from 1990 to 2019: Results from Global Burden of Disease study 2019
Source: PLoS One. 2022 Nov 14;17(11):e0277335. doi: 10.1371/journal.pone.0277335 (PMC9662722; doi:10.1371/journal.pone.0277335)
Supplement: S1 Table — (DOCX) [file pone.0277335.s004.docx]

| Supporting information 4 Table. Age-standardized DALY rate for every level 2 cause, 1990 to 2019 | | |
| --- | --- | --- |
|  | 1990 | 2019 |
| Cardiovascular diseases | 7619.9(6220.2-9154.5) | 7410.7(6281.3-8713.1) |
| Chronic respiratory diseases | 3444.2(2634.2-4285.7) | 2369.3(1964-2733.8) |
| Diabetes and kidney diseases | 1131.3(934.6-1342.7) | 1159.2(949.2-1404.8) |
| Digestive diseases | 1279.2(946-1675) | 917.6(643.7-1169.1) |
| Enteric infections | 393.7(254.4-589.9) | 255.3(174.1-360.6) |
| HIV/AIDS and sexually transmitted infections | 49.2(22.7-108.9) | 168.6(34.3-716.7) |
| Maternal and neonatal disorders | 3941.6(2971.6-5042.6) | 1175.9(936.3-1485.4) |
| Mental disorders | 1290.5(953.8-1701.5) | 1260.3(931.9-1658.9) |
| Musculoskeletal disorders | 1778.7(1285.6-2375) | 1766.2(1280.8-2333.4) |
| Neglected tropical diseases and malaria | 75.1(51.3-105.4) | 40.7(27.8-55.9) |
| Neoplasms | 4154.8(3269.8-5225.1) | 3546.2(2850.1-4344) |
| Neurological disorders | 1167.4(665-1915.6) | 1065.4(583.6-1790.8) |
| Nutritional deficiencies | 883.4(575.1-1280.6) | 380.9(256-542.1) |
| Other infectious diseases | 1782.9(1037.9-2899.8) | 208(163.3-272.2) |
| Other non-communicable diseases | 2670.2(1930-3530) | 1513.2(1153.6-1982.4) |
| Respiratory infections and tuberculosis | 3983.7(3097.8-5248.8) | 1214.5(1001.7-1464.5) |
| Self-harm and interpersonal violence | 976.7(741.8-1283.5) | 644.9(500.8-867.1) |
| Sense organ diseases | 758.3(508-1093.5) | 717.4(471.6-1045.9) |
| Skin and subcutaneous diseases | 631.9(419.7-917.1) | 621(407.4-910.5) |
| Substance use disorders | 301.2(231.6-380.4) | 298.3(227.5-376.2) |
| Transport injuries | 1872.3(1392.9-2515.6) | 1541(1164.4-2040) |
| Unintentional injuries | 2315.5(1808.3-2926.2) | 1167.6(925.1-1489) |
